# Supplementary material for: Enlarging the Scope of 5-Aminolevulinic Acid-Mediated Photodiagnosis towards Breast Cancers
Source: Int J Mol Sci. 2022 Nov 28;23(23):14900. doi: 10.3390/ijms232314900 (PMC9735814; doi:10.3390/ijms232314900)
Supplement: Supplementary file 1 [file ijms-23-14900-s001.zip › ijms-1913818-supplementary.pdf]

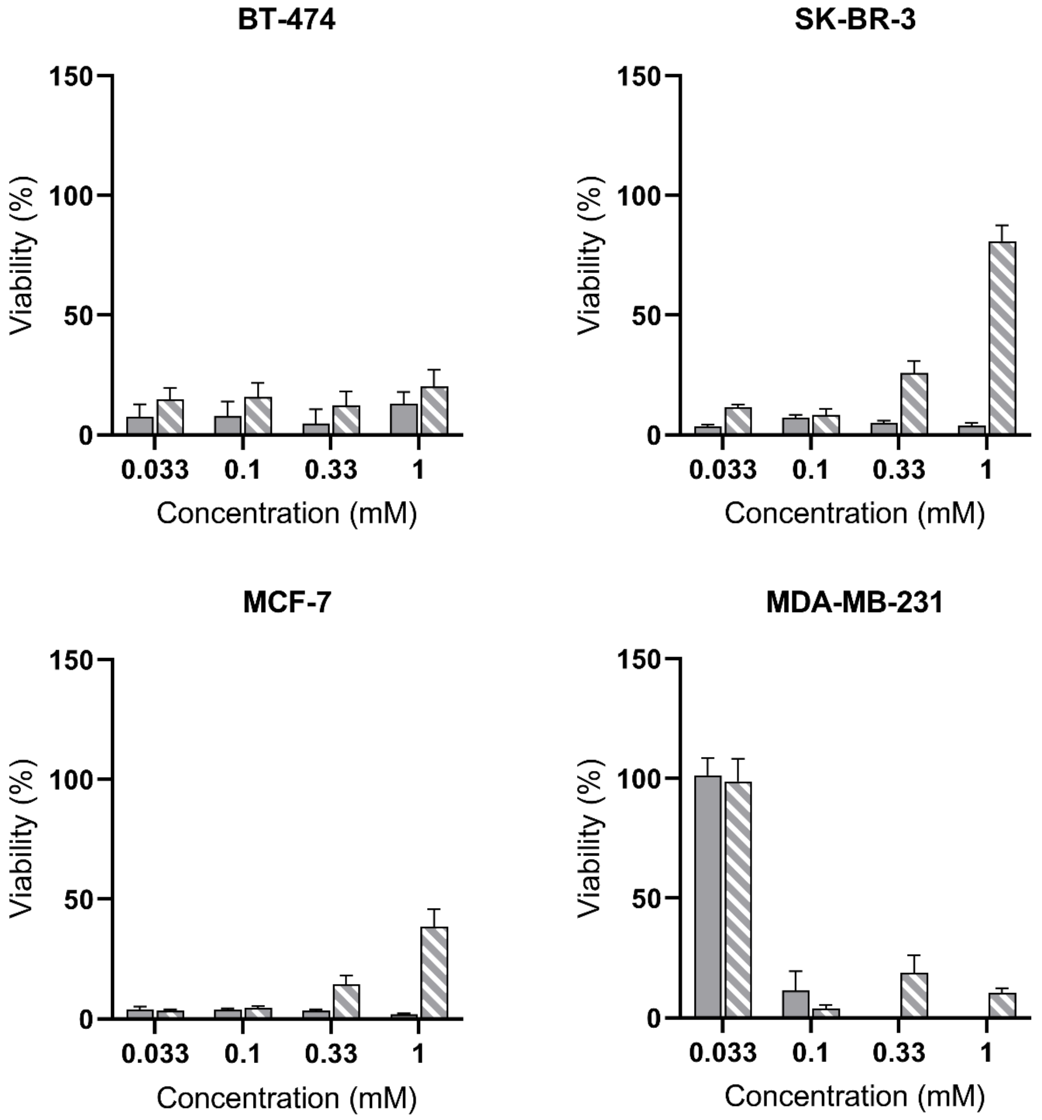

Figure S1. Evaluation of the photodynamic therapy potential of ALA-Hex (▬) and PSI-ALA-Hex (▨) at concentrations of 0.033, 0.1, 0.33 and 1 mM in BT-474, SK-BR-3, MCF-7 and MDA-MB-231 breast cancer cells in vitro. Cells were incubated for 6 h then exposed to blue light (10 J/cm^2) and PDT efficacy assessed 24 h post-irradiation by WST-1 viability measurement. Values are normalized against the untreated control exposed to the same blue light fluence.

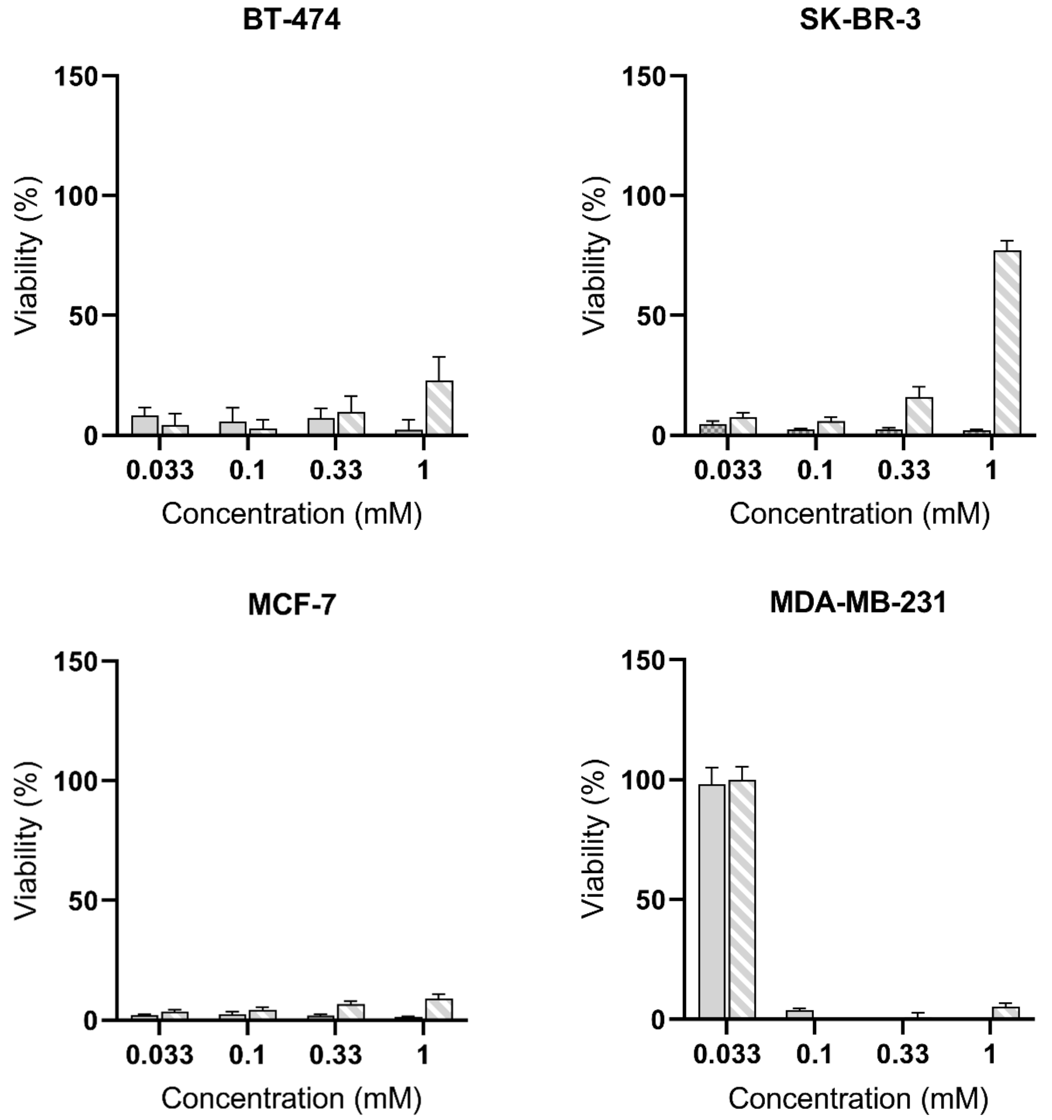

Figure S2. Evaluation of the photodynamic therapy potential of ALA-Hex (▭) and PSI-ALA-Hex (▨) at concentrations of 0.033, 0.1, 0.33 and 1 mM in BT-474, SK-BR-3, MCF-7 and MDA-MB-231 breast cancer cells in vitro. Cells were incubated for 6 h then exposed to blue light (20 J/cm^2) and PDT efficacy assessed 24 h post-irradiation by WST-1 viability measurement. Values are normalized against the untreated control exposed to the same blue light fluence.
